# “It’s Always the Judge’s Fault”: Attention, Emotion Recognition, and Expertise in Rhythmic Gymnastics Assessment

**DOI:** 10.3389/fpsyg.2016.01008

**Published:** 2016-07-05

**Authors:** Lindsey G. van Bokhorst, Lenka Knapová, Kim Majoranc, Zea K. Szebeni, Adam Táborský, Dragana Tomić, Elena Cañadas

**Affiliations:** ^1^Maastricht UniversityMaastricht, Netherlands; ^2^Masaryk UniversityBrno, Czech Republic; ^3^University of LjubljanaLjubljana, Slovenia; ^4^Eötvös Loránd UniversityBudapest, Hungary; ^5^University of Banja LukaBanja Luka, Bosnia and Herzegovina; ^6^University of LausanneLausanne, Switzerland

**Keywords:** rhythmic gymnastics, attention, emotion recognition, expertise, accuracy, judges

## Abstract

In many sports, such as figure skating or gymnastics, the outcome of a performance does not rely exclusively on objective measurements, but on more subjective cues. Judges need high attentional capacities to process visual information and overcome fatigue. Also their emotion recognition abilities might have an effect in detecting errors and making a more accurate assessment. Moreover, the scoring given by judges could be also influenced by their level of expertise. This study aims to assess how rhythmic gymnastics judges’ emotion recognition and attentional abilities influence accuracy of performance assessment. Data will be collected from rhythmic gymnastics judges and coaches at different international levels. This study will employ an online questionnaire consisting on an emotion recognition test and attentional test. Participants’ task is to watch a set of videotaped rhythmic gymnastics performances and evaluate them on the artistic and execution components of performance. Their scoring will be compared with the official scores given at the competition the video was taken from to measure the accuracy of the participants’ evaluations. The proposed research represents an interdisciplinary approach that integrates cognitive and sport psychology within experimental and applied contexts. The current study advances the theoretical understanding of how emotional and attentional aspects affect the evaluation of sport performance. The results will provide valuable evidence on the direction and strength of the relationship between the above-mentioned factors and the accuracy of sport performance evaluation. Importantly, practical implications might be drawn from this study. Intervention programs directed at improving the accuracy of judges could be created based on the understanding of how emotion recognition and attentional abilities are related to the accuracy of performance assessment.

## Introduction

Judges are often in the center of media coverage, repeatedly criticized, and sometimes undervalued part of the sport (Dosseville et al., 2014). Research concerning judges mostly focuses on the differences between novice and expert judges (Ste-Marie, 1999, 2000; Flessas et al., 2015) and the biases that occur during competitions (Ansorge and Scheer, 1988; Plessner, 1999; Plessner and Schallies, 2005). However, no attention has been devoted to psychological factors such as attention, emotion recognition of judges and possible interventions which could reduce biases or stress. Mentioned factors are relevant and may influence the outcome in sports with subjective scoring, such as rhythmic gymnastics, where the results – scoring and ranking of performances – depend heavily on evaluations made by judges.

Typically the scoring, as a main tool of evaluation, used in rhythmic gymnastics is based on subjective decisions of the judges. These decisions might be affected by cognitive abilities of judges such as selective attention and vigilance. Lower cognitive abilities affect the processing of visual information (Glisky, 2007). During a competition, judges have to track multiple objects at the same time which requires high demands on their resources. Research examined that individuals can identify multiple objects simultaneously, and in case of professional judges, the number is significantly higher (Cavanagh and Alvarez, 2005). It seems training and expertise increase cognitive resources available to process visual information. Oksama and Hyönä (2004) showed that the capability for tracking multiple objects can be affected by factors such as increased speed of the objects (which decreases capabilities) and the novelty of the objects (familiar objects increase capabilities).

Throughout the evaluation of rhythmic gymnastics performances, the judges have to follow the performance of five or six gymnasts at once, while each of the gymnasts also holds an apparatus (hoops, balls, or ribbons) which becomes separated from them during throws and other elements. While paying attention to bodily movements, the skilful movement of the apparatus, coordination, etc., judges face several distracting factors such as the color of the gymnasts’ dresses (Hagemann et al., 2008), the size of the crowd around them (Bai et al., 2007), the noise (Nevill et al., 2002), the disadvantageous angle from which they sometimes have to observe the performance (Oudejans et al., 2000), and even some psychological factors such as confirmation bias (Rodenberg, 2011).

Even though, rhythmic gymnastics settles rare conditions, it includes some human aspects which might be part of an evaluation and which cannot be avoided as emotions are present in every context of social interaction, including sport performance. They play an important part in the artistic evaluation of the performance, since the body movements and facial expressions have to be harmonized with the music. Emotion recognition is the ability to identify emotions of others through their nonverbal expression including face and body (Gabrielsson and Juslin, 1996). For gymnasts, emotions can influence sporting behaviors and actions through their effect on attention, and therefore concentration (Lazarus, 2000). Vast et al. (2010) studied the effect of perceived emotions on attention, especially concentration, and performance. They found that anger negatively affects concentration but not performance, whereas anxiety negatively affects performance but not concentration. Emotion recognition might play an important role during the evaluation of the performance in two ways. First, being able to accurately discriminate emotional intentions and expression of the gymnasts can improve assessment of the performance due to the amount of information that emotions provide about the situation and the mental state of the gymnasts. Second, emotions play an important role in error detection. For instance, a brief expression of anger or disappointment by the gymnasts can be an alerting signal to judges that a mistake was committed. Without emotion expression, the mistake could have passed unnoticed.

Not only emotions play an important role in evaluation. The expertise of judges has been found to influence the evaluation of performance of gymnasts. Ste-Marie (2000) found that novice judges spend less time looking at the gymnasts and more time looking at the scoring paper than the judges with more expertise. Flessas et al. (2015) confirmed that expert judges have better error detection than novices. However, even the highest ranked international judges only reported 40% of true errors. Other research suggests that expert judges are better at perceptually anticipating upcoming gymnastic elements, which has a positive effect on their judging performance as compared to novice judges (Ste-Marie, 1999). A study, done with football referees, obtained positive correlation indices among hours practiced per week, the number of competitions judged, and the ability to evaluate a performance (Catteeuw et al., 2009). Studies have also shown that both visual and motor experience with a specific sport account for correctly estimating the movement quality of other people (Loula et al., 2005; Blake and Shiffrar, 2007). However, according to a study concerning practical skills rhythmic gymnastics judges and their relation to the judging abilities was founded that judges mostly valued following skills: the knowledge of technical parameters of the sport and the capacity to adjust to any level of competition under self-assuredness and self-confidence circumstances, but not experience *per se* (Fernandez-Villarino et al., 2013).

Another factor that can greatly affect rhythmic gymnastics evaluation is chronotype of the judges. Chronotype is known as a trait that reflects individually preferred times for activityand sleep (Smith et al., 1989; Greenwood, 1994; Caci et al., 2000). According to the preferred times for activity, chronotypes are classified into “morningness”, “intermediate”, and “eveningness” types. Individuals of the morningness type prefer to be active in the early morning, whereas those of the eveningness type prefer to be active late in the evening. Chronotype is related to attention peaks at different times of day. Previous studies have shown that morning-types are more alert in the morning (Clarisse et al., 2010), while evening-types’ alertness did not differ between morning and afternoon (Adan and Guàrdia, 1993) or they performed better in the evening (Schmidt et al., 2012). However, some studies found no association between chronotype and performance inattention tests (Adan, 1991; Gomes et al., 2011; Adan et al., 2012).

Due to mentioned factors it might be beneficial to use the Rasch model as it has been previously reported to be beneficial in assessing sport performances, and more concretely to evaluate consistency among the different evaluations of a judge and the different aspects of performance been evaluated (Looney, 2003).

Previous research on judged scoring in sports has mostly focused on the differences between novice and expert judges, but research on psychological factors (i.e., attention and emotion recognition) and their influence on performance assessment have yet to be investigated. The present study investigates how attentional and emotion recognition abilities can affect accuracy of sport assessment in rhythmic gymnastics. The primary goal of this study is to evaluate attention and emotion recognition in rhythmic gymnastics judges and their relationship with accuracy in performance evaluation. Furthermore, the aim is to investigate possible differences between novice and expert judges. Finally, the results could help in improving the accuracy and future training of judges. We hypothesize that (i) attentional and emotion recognition abilities of judges are positively correlated with scoring accuracy, (ii) higher level of expertise is positively correlated with scoring accuracy, and (iii) expertise has a mediating role between attentional and emotion recognition abilities of the judges and scoring accuracy.

## Materials and Methods

### Materials and Measures/Equipment

#### Video

Participants will evaluate a set of videotaped group rhythmic gymnastics performances (2–3 min each) on the artistic and execution components of the performance score. Using recordings of past performances will ensure the same conditions for all participants and that will allow us to compare the differences in scoring between participants. Detailed information about the selected videos can be found in **Table 1.** These videos were selected in a two-step process. Firstly, all the videos from past Olympic Games freely available online were gathered. The Olympic Games were chosen for its best video coverage as well as broadest competition. It was decided that videos from the latest 2012 Olympic Games should be used with respect to the recording quality that ensures better visibility of performance details that is crucial for accurate performance evaluation. In the second step, the videos were studied regarding the obtained score, noticeability of errors, and dress conspicuousness. In the end, four videos including a range of performances from almost perfect performances to performances with more noticeable errors were selected. The resolution of the videos is 720p and the sound is muted to prevent the participants’ evaluations from being influenced by the commentary.

**Table 1:** Chosen group rhythmic gymnastics performances from 2012 Olympic Games.

Competition	Discipline	Nation	Score artistic	Score execution	Place
Qualification	3 hoops, 2 ribbons	Russian Federation	9.550	9.100	1st
Qualification	3 hoops, 2 ribbons	Greece	8.900	8.450	9th
Final	3 hoops, 2 ribbons	Spain	9.300	8.800	4th
Final^a^	3 hoops, 2 ribbons	Bulgaria	9.450	8.850	6th


*^a^This video will be used as a practice video to get familiar with the judging process.*

A certain risk to the study is the fact that judges might remember the scores of the performances that were given at the Olympic Games. To check for this, the question: “Have you seen this performance before?” will be asked after each video. If their answer is yes, then they will be asked to write down the remembered scores. This will allow us to see whether they actually remembered the score or whether the performance just looked familiar to them and control for this in the analysis.

#### Attentional Abilities

**FIGURE 1:**
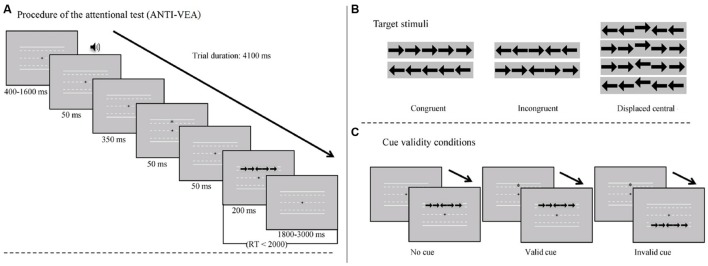
**Attention Network Test for Interactions and vigilance of activation (ANTI-VEA): **(A)** Schematic representation of the procedure. **(B)** Target stimuli. **(C)** Visual cue conditions.** Adapted from “Measuring vigilance while assessing the functioning of the three attentional networks: The ANTI-Vigilance task,” by Roca et al., 2011, *Journal of Neuroscience Methods, 298*(2), p. 316. Copyright 2015 by Elsevier B.V. Reprinted with permission.

The attentional abilities will be measured by the Attention Network Test for Interactions and Vigilance (ANTI-V; Roca et al., 2011; including new measures of vigilance executive and vigilance of activation – ANTI-VEA) that takes about 15 minutes to complete. The test (**Figure 1**) is computer-based and measures participants’ performance in five components of attention: phasic alertness, vigilance executive, vigilance of activation, orienting network, and executive control. ANTI-VEA measures these components through reaction time and accuracy (or percentage of errors). Participants have to indicate the direction of the central stimulus, by pressing C or M on the keyboard (typical flanker task). Direction of the flanker stimuli (either congruent or incongruent) tests the executive control functioning. When participants detect that the central stimulus is displaced, they have to press spacebar. This type of stimuli is unpredictable, infrequent, and unexpected and detection of such a stimulus measures vigilance executive, as Interactions and Vigilance (ANTI-V; Roca et al., 2011). Vigilance of activation is measured by the Psychomotor Vigilance Task (PVT; Correa et al., 2014), a numerical count down starting at 999 ms that participants have to stop as fast as possible by pressing any key. Visual cue assesses the functioning of the orienting network (valid, invalid, or no cue condition). Auditory warning signal (50 ms) that announces the appearance of the stimulus measures phasic alertness.

The advantage of ANTI-VEA is that it tests the five attentional components in an independent way and allows measuring of the interactions between them. Moreover, it measures the reaction time for every answer. Reliability of ANTI-V was 0.99 (Roca, 2012) and we expect the ANTI-VEA to be highly reliable as well as it is a new, slightly adjusted, version of ANTI-V.

#### Emotion Recognition Ability

The Geneva Emotion Recognition Test-Short (GERT-S; Schlegel and Scherer, 2015) measures individual differences in the ability to recognize emotions of others. It is a shortened version of the Geneva Emotion Recognition Test (GERT; Schlegel et al., 2014). GERT-S consists of 42 items short audio–visual clips in which ten actors express 14 different emotions (e.g., anger, relief, joy, pride, and anxiety) through their face, voice, and body. After each clip, participants have to choose one of the 14 emotions which they believe was expressed by the actor. This test is dynamic and multimodal (short video clips with sound) and measures ERA in a more ecologically valid fashion. In comparison with other ERA tests which usually offer basic emotions, GERT-S features 14 different emotions, both positive and negative. Moreover, GERT-S internal consistency is between 0.80 and 0.83 (Schlegel and Scherer, 2015).

#### Expertise

Participants will be asked about their judging experience (judging category, years of judging at the international level, and number of judged competitions) to determine their expertise. There are four categories of FIG judges for all disciplines, Category IV being the lowest and Category I the highest. Category IV Judges are new international judges with no or little international experience. They are not assigned as judges in major competitions. Category III Judges are defined as experienced judges with good results in execution and artistic. They are designated to judge execution and artistic in major competitions. Category II Judges are experienced judges with very good results in difficulty. They are designated to judge difficulty as well as execution and artistic. Category I Judges are very experienced judges with excellency in difficulty and operate as members of the Superior Jury, Chair of Judges’ Panel, and Difficulty Judges and are also designated to judge execution and artistry (Fédération Internationale de Gymnastique [FIG], 2013).

#### Additional Questions

Participants will further answer basic demographic questions (gender, age, nationality, etc.), questions about their chronotype (rMEQ; Adan and Almirall, 1991; example question: *At what time in the evening do you feel tired and as a result in need of sleep?*), and manipulation check questions (whether they have seen the presented performances before and whether they remember the scores).

## Stepwise Procedure

### Power Analysis

The software G^∗^power (Faul et al., 2007) was used to calculate the required sample size. Values for α were set on 0.05 and power on 0.80. Based on previous literature and discussions between the authors, effect sizes were estimated on 0.15 (medium effect). In total, to reach the desired power, data from 55 participants has to be collected.

### Qualtrics Survey

The study uses an online survey designed on Qualtrics which enables judges from different countries to participate. There is a great benefit of using videotaped performances that can be assessed on any computer over real-time evaluations at competitions. With regards to the different nationalities of the participants, the instructions will be translated into several languages to optimize the comprehensibility of the survey for judges from different cultural backgrounds and native languages. Although translating instructions into different languages might pose limitations to the internal validity of the study, the current study will use native speakers with a fluency in English to translate instructions into their native languages. Participants recruitment and the overall small research population can also cause a limitation in regards to the sample size of the research, but it is important to notice that the actual population of judges in rhythmic gymnastics at international level is also limited (376 judges approved by the FIG). Therefore, it would be interesting in future experiments to include judges from different disciplines, such as dance sport, to extent the research population. Finally, the current study applied a new approach to measure accuracy of performance scoring, as to the authors’ knowledge such measuring tools have not been developed nor validated. However, the official scores given at international competitions are considered as the most accurate attainable scores, and it can therefore be concluded that participants that gave similar scores have high scoring accuracy.

### Participant Recruitment

This study aims to recruit 50 rhythmic gymnastics judges approved by the International Gymnastics Federation (13% of the total number of judges at this level) and a control group of 20 rhythmic gymnastics coaches unskilled in performance judging. Participants will be recruited via email, social media, personal contact, and with the help of national gymnastics federations. After confirming participation via a consent form following the ethical committee approval from the University of Lausanne, they will complete an online testing session. Participation will be anonymous and voluntary.

### Procedure

Data will be collected using an online survey design based on Qualtrics including measures of personal characteristics and the assessment of videotaped performances of rhythmic gymnasts. The survey will have multiple language versions. The first part of the survey will focus on the evaluation of group rhythmic gymnastics performances. Initially, participants will be asked to read the International Gymnastics Federation’s Code of Points on the artistic and execution components. Participants will then be given one practice video to get familiar with the judging process. After the practice video, participants will be shown three video clips of performances from the 2012 Olympic Games. Participants will watch the videos and score them according to the artistic and execution components. Their scores will be compared to the official scores given at the competition the videos were taken from to determine the scoring accuracy of each participant. The official scores given at the Olympic Games are considered to be the most accurate scores that are attainable for this purpose. The second part of the online survey will consist of two tests measuring attentional (ANTI-VEA) and emotion recognition abilities (GERT-S). Finally, participants will answer additional questions about their judging experience (expertise), chronotype (rMEQ), demographic data, and manipulation check.

## Statistical Analysis

To test our hypothesis we will use statistical software including SPSS, R and Stata. We will follow the four-faceted Rasch model (gymnastic group ability, aspect difficulty, program difficulty, and judge severity) to assure that valid evaluation occurred between videos and within judges. To do that, we will follow the protocol recommended by Linacre (1999) and Wright and Mok (2000) by mean of the Mififac Rasch Software. Once confirmed that the data fit the model well enough so judges’ performance evaluations are valid, we then will create a variable that indicates how much actual judges’ evaluations deviate from Olympic judges’ evaluations (accuracy).

**FIGURE 2:**
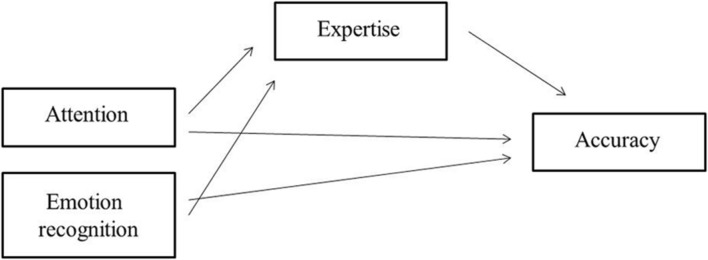
**Proposed model of the relation between expertise and scoring accuracy**.

Next, we will perform correlational analysis to evaluate the hypothesized positive correlations between attentional abilities and accuracy, ERA and accuracy as well as expertise and accuracy. When testing a more complex model that includes causality and interrelations between the variables, we encounter the problem of working with endogenous variables, in our case latent variables that are being estimated through their manifestations, i.e., attention and emotion recognition (Antonakis et al., 2010). Moreover, the relationship between these variables and the dependent variable (accuracy) is expected to partly function through other variables in the model (i.e., expertise). Therefore the data will be further analyzed using regression analysis and Structural Equation Modelling (SEM) to test a mediational role of the relationship between expertise and accuracy (**Figure 2**).

The evaluation of the fit of the model to the obtained data we will rely on the Root Mean Square Error of Approximation index (RMSEA; Steiger and Lind, 1980) that is based on the χ2 value of the model as well as the number of observed cases and degrees of freedom, as depicted in Equation 1. RMSEA’s values of 0.05 and lower indicate a good fit, while values up to 0.08 indicate an acceptable fit (Browne and Cudeck, 1993). We will use also *SWAIN* correction, as a measure to address sensitivities of χ^2^ statistic to sample size and model complexity (Antonakis and Bastardoz, 2013). RMSEA formula retrieved from http://davidakenny.net/cm/fit.htm,

$$\frac{\sqrt{\left(\chi^{2}-df\right)}}{\sqrt{\left[df\left(N-1\right)\right]}}$$

To address the above-mentioned issue of endogeneity and to deliver consistent parameter estimates, the analysis will follow Antonakis et al. (2010) recommendations: (a) include instrumental variables (variables uncorrelated with the error term of the dependent variable and correlated with the endogenous variables) that are necessary to provide a sufficient identification of the model, for instance, age, gender, etc., (b) be performed through the Two-Stage Least-Squares procedure (2SLS; James and Singh, 1978; Baltagi, 2011), a tool used in SEM to estimate path coefficients. In a first-stage, attention and ERA will be regressed on two instrumental variables and covariates, including fixed effects. In the second-stage, accuracy will be regressed on the predicted value of attention and ERA from the first stage and covariates, including fixed effects. We will verify that the instrumental variables used are supported by theoretical and empirical considerations.

## Anticipated Results

We expect to support our hypotheses by testing the model presented in the methods section. The first hypothesis stated that attention and emotion recognition are positively related to scoring accuracy. It is assumed that higher levels of attention improve error detection of judges and therefore enable them to rate the performances more accurately, and higher levels of emotion recognition provide judges with the skill to detect changes in facial or bodily expressions that accompany mistakes in the performance. The second and third hypotheses propose a relationship between expertise and attention and emotion recognition and a relationship between expertise and scoring accuracy. By testing our anticipated model we expect to find that expertise is also associated with attention and emotion recognition, and expertise itself is associated with scoring accuracy. Better expertise, defined both as level and years of judging, provides judges with more experience and training to judge more accurately, and it may therefore be assumed that judges of higher levels would be significantly more accurate in their assessments.

The study is facing some potential pitfalls when collecting data. For example, as participants include judges at international levels, some may have already seen the performances to be evaluated in the survey and therefore remember the scores that were given at the competition. We will control for positive answer to those questions. Another important factor that is included in the survey is whether participants are morning or evening types (i.e., chronotype), as different types reach their peak level of attention and concentration at different moments during the day. Not completing the survey within one’s peak hours may possibly influence the person’s accuracy and scores of the ANTI-VEA negatively. To take this into account, we control for both self-reported chronotype as well as the moment of the day the tasks were performed, to make sure everyone was at their full activity range.

## Author Contributions

EC is the author of the concept of the study, the supervisor of the project, and provided feedback when writing the manuscript. KM, ZS, and AT were in charge of writing the introductory part of the manuscript (including literature review). LvB, LK, and DT were in charge of writing the Materials and Methods, Stepwise Procedure, Statistical Analysis, and Anticipated Results. All of the authors contributed to frequent feedback sessions and the development of the project in general.

## Conflict of Interest Statement

The authors declare that the research was conducted in the absence of any commercial or financial relationships that could be construed as a potential conflict of interest.
